# Editorial: Advances in the treatment of primary central nervous system lymphoma

**DOI:** 10.3389/fonc.2023.1271566

**Published:** 2023-08-14

**Authors:** Liren Qian, Julio C. Chavez, Gaurav Prakash

**Affiliations:** ^1^ Senior Department of Hematology, The Fifth Medical Center of PLA General Hospital, Beijing, China; ^2^ Department of Malignant Hematology, Moffitt Cancer Center, Tampa, FL, United States; ^3^ Department of Clinical Hematology and Medical Oncology, Postgraduate Institute of Medical Education and Research, Chandigarh, India

**Keywords:** central nervous system, diffuse large B-cell lymphoma, PCNSL, autologous stem-cell transplantation, chimeric antigen receptor, CD79b

Primary central nervous system lymphoma was first reported by Percival Bailey in 1929, but he did not explicitly name it “lymphoma”, but considered this type of tumor to be a perivascular sarcoma, originating from the perivascular sheath of the leptomeninges (1). In 1938, Yuile C. L. described it as “primary reticulum cell sarcoma of the brain” by a case report (2). Until 1963, Bursrein, S. D. et al. found that reticuloendothelial neoplasms of the central nervous system were histologically similar to lymphoma (3). Nevertheless, they included this type in the category of reticulum cell sarcoma. In 1974, James M. Henry et al. found that primary “reticular cell sarcomato-microgliomatosis” of central nervous system was histologically identical to malignant lymphoma, so it was named primary central nervous system lymphoma (PCNSL) (4).

After nearly a century of development, great progress has been made in the diagnosis, classification, staging, treatment and prognosis of PCNSL. The ultimate goal of these basic and clinical researches is to prolong the life of patients and improve their quality of life. This Research Topic focuses on the advances in the treatment of PCNSL, wish to improve the prognosis of this rare central nervous system tumor. This Research Topic contains 5 articles, involving chimeric antigen receptor T(CAR-T) cell therapy, autologous hematopoietic stem cell transplantation, new chemotherapy regimens and new prognostic models.

In the article by Miyao et al. from Japanese Anjo Kosei Hospital, they reviewed the role of Chimeric antigen receptor T (CAR-T) cells in PCNSL. CAR-T cell therapy has been proved to be effective in peripheral B-cell lymphoma, myeloma and other hematologic malignancies, but its role in PCNSL is still under clinical study. This review highlighted current state of CAR-T cell therapy in PCNSL, its resistance mechanisms and future directions in the field. It was suggested that the response rate and durable response of PCNSL could be improved by CAR-T cells combined with conventional high-dose MTX chemotherapy. Combining CAR-T cells with conventional high-dose MTX chemotherapy could also improve the response rate and durable response for PCNSL, and reduce the dose of conventional high-dose chemotherapy and its toxicity.

Liu et al. from the Fifth Medical Center of Chinese PLA General Hospital attempted to identify the status of autologous stem-cell transplantation (ASCT) by meta-analysis. The status of ASCT in the PCNSL is still controversial. By analyzing data from PubMed, Embase, and Cochrane Library databases on newly diagnosed PCNSL, they found that consolidation with ASCT after chemotherapy may achieve better therapeutic outcomes: complete remission rate, overall response rate, and relapse rate were 80%, 95% and 19%, 5-year PFS and OS rates were 65% and 69%, comparable to whole brain radiation. Most of the current studies on the application of ASCT in PCNSL are single-arm studies or retrospective studies. Based on this meta-analysis, we expect further randomized double-blind controlled studies with a large sample size and higher evidence level.

Sun et al. from Chinese Beijing Tiantan Hospital analyzed 243 patients with PCNSL retrospectively. They found that among 95 patients receiving non-myeloablative sequential consolidation chemotherapy by either pemetrexed or etoposide plus cytarabine, median PFS was 28 months, and estimated 4 years overall survival was 78.7%, higher than 14 months and 61.6% for all patients. Age and early treatment response were independent predictors of survival. It was suggested that high-dose MTX induction chemotherapy with sequential non-myeloablative consolidation chemotherapy may be a better treatment for PCNSL. Compared with the results in the article by Liu et al. in this Research Topic, non-myeloablative sequential consolidation chemotherapy was not inferior to ASCT. However, more rigorous prospective controlled studies are still needed for further demonstration.

In the article by Zhou et al. from Chinese Shenzhen People’s Hospital, primary CNS diffuse large B cell lymphoma was divided the into CDP group and non-CDP group. The biggest difficulty of targeted precision therapy in DLBCL is the heterogeneity of genetic characteristics and phenotypes of DLBCL. It is now clear that DLBCL is not a single disease, but a series of subgroups of diseases, each with distinct molecular and biological characteristics and dependent on different carcinogenic pathways. This biological heterogeneity divided DLBCL into more subgroups by genotyping. In 2000, Alizadeh et al. divided DLBCL into GCB type and ABC type (5). In 2002, Rosenwald et al. divided DLBCL into GCB type, ABC type and Type 3 (6), opening a chapter of precise treatment of lymphoma. Since then, with the improvement of detection technology, the genotyping of DLBCL has made rapid progress until the 5 classification by Chapuy et al. in 2018 (7), the 7 classification in 2020 and 2021 by National Cancer Institute (NCI) and UK Hematological Malignancies Research Network (HMRN) (8–10). These genotypes provided a reliable basis for the precise treatment of DLBCL. It is not clear whether the primary CNS diffuse large B cell lymphoma is consistent with the gene subtype of peripheral DLBCL. This study divided PCNSL patients into CDP group and non-CDP group based on whether they had CD79B and PIM1 mutations. They found that CDP patients had a higher 2-year survival rate than non-CDP patients (76% VS 40%). Further research is needed to confirm the exact mechanism and whether primary CNS DLBCL can be further subdivided like peripheral DLBCL. Perhaps this study will open a new chapter of PCNSL precision therapy.

A novel prognostic model of PCNSL was established based on the systemic immune-inflammation index (SII) and the Memorial Sloan Kettering Cancer Center (MSKCC) model by Li et al. from Chinese Huashan Hospital. The MSKCC model has been used to predict the prognosis of PCNSL since it was reported in 2006. In recent 2 decades, the diagnosis, treatment and prognosis of PCNSL have made great progress, so the model needs to be updated. In addition, SII has also been used to predict the prognosis of various malignant tumors, and has also been used to predict the prognosis of PCNSL. In this paper, a new SII-MSKCC prognostic model (including SII, age, and KPS score) was established and validated by combining SII and MSKCC models. It was found that SII-MSKCC model is more precise than MSKCC model, which needs to be further verified in clinical practice and may be useful for clinical application.

In conclusion, this Research Topic brings together the latest cutting-edge researches on the treatment and prognosis of PCNSL, which makes a contribution to the development of the treatment of PCNSL and points out the direction of further research. Finally, we would like to thank the authors for publishing their excellent papers on our Research Topic, as well as the reviewers for their professional opinions and the editorial staff for their support.

## Author contributions

LQ: Conceptualization, Writing – original draft, Writing – review & editing. JC: Writing – review & editing, Resources. GP: Resources, Writing – review & editing.
